# Infants’ and Children’s Salt Taste Perception and Liking: A Review

**DOI:** 10.3390/nu9091011

**Published:** 2017-09-13

**Authors:** Djin G. Liem

**Affiliations:** Centre for Advanced Sensory Science, School of Exercise and Nutrition Sciences, Deakin University, 221 Burwood Highway, Burwood, VIC 3125, Australia; gie.liem@deakin.edu.au; Tel.: +61-03-9244-6039

**Keywords:** taste, smell, salt, foods, nutrition, children, sensory, intake, development

## Abstract

Sodium is an essential nutrient for the human body. It is widely used as sodium chloride (table salt) in (processed) foods and overconsumed by both children and adults, placing them at risk for adverse health effects such as high blood pressure and cardiovascular diseases. The current review focusses on the development of salt taste sensitivity and preferences, and its association with food intake. Three -to- four month old infants are able to detect and prefer sodium chloride solutions over plain water, which is thought to be a biological unlearned response. Liking for water with sodium chloride mostly decreases when infants enter early childhood, but liking for sodium chloride in appropriate food contexts such as soup and snack foods remains high. The increased acceptance and preference of sodium chloride rich foods coincides with infants’ exposure to salty foods, and is therefore thought to be mostly a learned response. Children prefer higher salt concentrations than adults, but seem to be equally sensitive to salt taste. The addition of salt to foods increases children’s consumption of those foods. However, children’s liking for salt taste as such does not seem to correlate with children’s consumption of salty foods. Decreasing the exposure to salty tasting foods during early infancy is recommended. Salt plays an important role in children’s liking for a variety of foods. It is, however, questionable if children’s liking for salt per se influences the intake of salty foods.

## 1. Introduction

Sodium is essential for the regulation of the osmotic pressure and extracellular fluids in the human body. It has been estimated that humans need about 180 to 230 mg of sodium per day for normal bodily functioning [1]. Sodium cannot be produced by the human body and therefore needs to be ingested, which, in modern society, is done so mostly in the form of sodium chloride (i.e., table salt). Despite the biological need for sodium, excessive sodium consumption has been related to a range of adverse health outcomes, such as hypertension, gastric cancer and obesity [2,3,4]. Therefore, the WHO has recommended for adults to consume no more than 2 g of sodium per day. For children (2–16 years) this limit is adjusted downwards, based on energy requirements [5]. In Australia the National Health and Research Council recommends an increasing upper limit with age starting with 1 g of sodium/day as the upper limit for 2–3 years old, which should not exceed 2.3 g sodium/day when children enter the adolescence years (14–18 years) [6]. This is similar to upper limits published by the Center for Disease Control in the United States of America (US) [7].

Sodium chloride is a relatively cheap and widely used ingredient in processed foods [8] and serves a variety of functions. The addition of salt limits microbial growth by lowering the water activity [9]. Texture and juiciness of foods are enhanced by the interaction of salt with protein, and by the enhancement of the hydration and water holding capacity of foods [10]. In bakery products, salt strengthens the gluten network, which improves the elasticity of the dough [11,12]. Moreover, salt is added to foods to improve the flavour profile. The addition of salt not only increases saltiness, but also suppresses bitterness. When bitterness is decreased the sweet taste of food is more noticed, resulting in a generally liked flavour profile [13]. As a result of the wide spread use of sodium chloride in processed foods and the reliance of modern consumers on processed foods, the majority of sodium (75%) in the Western diet comes from processed foods and restaurant foods [14,15]. Only a small proportion of total sodium intake comes from natural sources and from what consumers add to their food during preparation and consumption (i.e., 10 to 15%) [9].

The biological need for sodium, combined with a scarcity of sodium in the diet of the primate ancestors of humans, likely resulted in an evolutionary human drive to consume sodium [16]. Nowadays, the wide spread use of sodium chloride in processed foods has made sodium extremely accessible. This, not surprisingly, leads to an overconsumption of sodium. Yet, it is important to note that well before the existence of modern processed foods, sodium consumption has been well above (i.e., 3–5 g of sodium/day) the human physiological requirements [15]. The consistency in intake over time and across different ethnic populations suggest that the modern food industry is not the only factor involved in humans’ high sodium consumption and that unknown physiological or nutritional factors might be involved.

The excessive consumption of sodium by adults and children is, nowadays, mostly derived from sodium chloride. In a large study including 187 countries it was found that 99.2% of the adult population consumed more sodium than the WHO’s recommended upper limit of 2 g of sodium per day. The vast majority (88.3%) of the adult population consumed more than 3 g of sodium per day [3]. An Australian study found that sodium intake rapidly doubled in the first 2 years of life, to an estimated 1 g of sodium per day by the age of 17 months [17]. By the age of 4 years the average sodium intake has been estimated at almost 1.5 g of sodium per day [18]. Data from the US [19] suggested that children’s sodium intake further increases with age (6–13 years: 3.1 g/day, 14–18 years: 3.6 g/day). Such an increase can partly be explained by an increased total food consumption with age, but can also be a consequence of an increased sodium density of children’s diets [19]. It has been estimated that about half of the sodium children in the US consume comes from only 10 food categories that include pizza, Mexican-mixed dishes, sandwiches, breads, cold cuts, soups, savoury snacks, cheese, plain milk, and poultry [19]. This is in line with 9- to 13-year-old children in Australia (mean sodium intake = 2.7 g/day), who consumed most of their sodium from cereal based products, and meat and poultry products [20]. The problem with these high levels of sodium consumption at a young age is two-fold. Firstly, it sets children up for developing high blood pressure during childhood and adulthood [21,22]. Secondly, children might get used to eating high levels of salt, and expect a certain level of saltiness in their foods. This potentially leads to unhealthy food choices during child-and adulthood.

This signifies the need to understand why infants and children consume such high amounts of sodium, which far exceeds their biological need. In this quest it is worth investigating how the ability to sense sodium and a liking for salty foods develops, given that food-liking plays a key role in children’s [23] and infants’ [24] food choice and consumption. This review aims to provide an overview of the current knowledge about the development of salt taste perception and salt taste liking during infancy and childhood and its relationship to food consumption and health outcomes. This review focusses on human research in non-clinical populations and will not provide an extensive review of neurological processes and brain structures involved in salt appetite in severely sodium deficient populations or animals.

## 2. Literature Search

A systematic literature search was conducted as of 6 June 2017 using Medline Complete, PsycINFO, Social Sciences Citation Index, Scopus, Psychology and Behavioural Science Collection, Cochrane Database of Systematic Reviews and Emerald Insight. The following search terms were used: (taste OR preference OR sensory) AND (salt OR sodium) AND (infants OR baby OR newborn OR neonate). This resulted in 330 references. A complete manual search of the reference lists of original studies was also conducted. Studies which did not have salty taste preferences or perception as either a dependent variable or independent variable, or were conducted in only adults or animals were excluded. After excluding duplicates, and irrelevant titles, 54 references were considered relevant for the present review.

## 3. Taste Perception

When salty foods or beverages are tasted, both physiological and cognitive related processes take place. Salt taste perception is derived from the interaction of sodium with amiloride-sensitive epithelial sodium channels (ENaC) in the human taste buds on the tongue [9]. In addition ENaCs have been located in the distal nephron, distal colon and airway epithelia, where they play an important role in Na^+^ reabsorption [25]. This suggests a potential link between salt taste perception and other (e.g., renal) functions in the body [26]. Furthermore, it has been suggested that salt taste perception through the interaction of sodium with ENaCs is not the only mechanism involved in human salt taste perception. Additional cellular and molecular mechanisms are not yet understood fully [15].

Neurological signals which result from the interaction between sodium and sodium sensing channels are transmitted to the brain and interpreted in specific parts of the brain. A strong enough signal results in the perception of a variety of taste related aspects such as detection, intensity, taste quality (e.g., sweet, sour, bitter, salty, umami), and hedonics [27].

Humans’ ability to detect sodium, which is part of taste sensitivity, mainly represents the physiological process of taste perception, whereas hedonics (e.g., liking, preference, acceptance) is a result of the cognitive interpretation of the physiological taste signals. Although both are part of taste functioning, the underlying principles and methods on how to assess these are different. It is generally found that taste sensitivity and taste hedonics do not correlate well [28], emphasising that both represent different pathways. This makes it important to investigate both taste sensitivity and hedonics, in order to understand the relationship between taste functioning and health outcomes.

Many animals, including humans, are able to sense sodium in the food supply by taste [29], however not all sodium in foods can easily be tasted [30]. For example, sodium in bread can be reduced to some extent without children [31] nor adults [32] noticing the difference. Sodium chloride is the only food grade chemical which elicits a pure salt taste [26]. More precisely, it is the positively charged sodium ion, rather than the chloride anion, which is mainly responsible for salty taste [33]. Other salts such as potassium chloride elicit a salty taste, but often taste bitter when used in high quantity. Lithium chloride does elicit a pure salt taste, but cannot be used in foods because of its toxicity (for review see [34]). In the absence of effective salt replacers, sodium chloride (hereafter referred to as “salt”) is the only chemical which in practice can be used to make food taste saltier.

Humans have a high liking for salty tasting foods, which, from an evolutionary point of view, would result in humans preferring foods with sodium, which is needed for survival. The addition of salt to food changes the complete sensory profile, beyond making a food taste saltier. In both children [35] and adults [13,36], it has been shown that the addition of salt to food can make the food taste less bitter. Such suppression of bitter taste can increase perceived sweetness of particular foods [36]. Salt is mostly consumed in the contexts of specific foods, rather than pure salt itself. Therefore, the measurement of salt taste sensitivity is usually carried out with salted water, whereas the measurement of children’s hedonic response to salt in foods is usually carried out with stimuli in which salt is deemed appropriate such as soups, broth, and crackers.

## 4. Salt Taste Detection and Acceptance in Infants

Infants’ ability to detect salt taste is mainly measured by the quantity the infant ingests, sucking patterns and facial expression in response to ingestion of water solutions with different concentrations of salt [37,38]. Because of the nature of these measurements it is hard to distinguish between infants’ ability to detect sodium and infants’ preference for, or liking of, sodium.

With these limitations in mind, it has been found that infants go through different developmental stages of acceptance of salted water and salted foods. When newborns (1–4 days old) are presented with 4.3 g of salt/100 mL of water, facial expressions suggest an indifference to salty taste [37,38]. This is unlikely to be due to the newborns’ inability to respond to taste per se. Facial and ingestive responses to bitter, sweet and to some extent sour taste, suggest that newborns can discriminate between different taste stimuli [37,38,39,40]. Animal studies suggest that the specific central and/or peripheral mechanisms, underlying salt taste perception, mature postnatally [41]. In other words, newborns might not be able to detect salt taste until further maturation of the salt taste system. It needs, however, to be mentioned that one study observed that newborns decrease their sucking burst frequency in response to mild salt tasting solutions (0.58 g of salt/100 mL of water) [42]. It has been hypothesised that such signs of lower acceptance (compared to water) is caused by the interaction of sodium with taste fibres which are not specific to sodium and might therefore elicit other taste responses such as slight bitterness, which infants reject [43].

Newborns have a biological need for sodium, which brings up the question of why newborns are not able to show a preferential response to salty taste, like they show for sweet taste [38]. The lack of a salt taste response might have an evolutionary reason. Although there is a need for sodium at birth, the first food the infant naturally encounters is breastmilk, which is dominated by sweet taste [44] and contains a sufficient concentration of sodium for the baby to thrive [45]. Children have an inborn preference for sweet taste, which would lead to a natural acceptance and consumption of breastmilk. This makes an inborn acceptance of salt taste, from an evolutionary point of view, not needed per se.

By the age of approximately 4 to 6 months, when sodium channels have further matured, infants show a preference for salty water over plain water [38,46], and salted baby cereal over plain baby cereal [47], as measured by ingestion. This shift from indifference to preference of salted water is thought to reflect an unlearned biological response to salty taste, rather than a learned response [48]. This does not mean, however, that the addition of salt to any baby food would ensure an increase in consumption. When salt was added to baby formula, 6- to 7-months-old infants found it less palatable, as measured by frequency of sucks, than baby formula without added salt [43]. Presumably because the addition of salt made the baby formula less sweet, which was confirmed by a trained adult sensory panel [43]. Alternatively, but not mutually exclusively, the addition of salt created an unknown flavour combination, which infants rejected because of its novelty.

In summary, infants’ ability to detect salt taste develops postnatally such that infants younger than about 3 months of age are most likely not able to detect salt taste. Once infants can detect salt taste they show a preference for salt taste in water. There is no prior exposure to salt taste needed for infants to prefer salted water, which suggests an unlearned biological response to salt taste.

**Figure 1:**
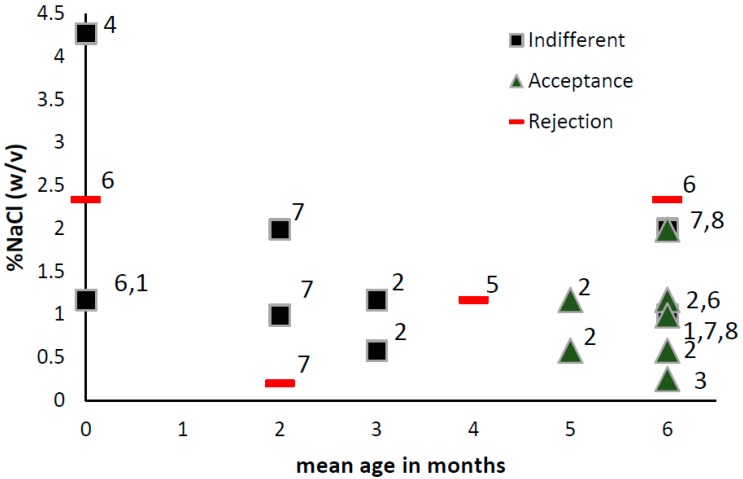
Infants’ (0–6 months) Indifference, acceptance and rejection responses to different concentrations of NaCl water (%NaCl *w*/*v*). Age on the y-axis represent mean age in months. 1 Maller & Desor 1973, measure: intake, stimuli: salt water [40]; 2 Beauchamp,Cowart &Moran 1986, measure: intake, stimuli: salt water [49]; 3 Harris & Booth 1987, measure: intake, stimuli: cereals [50]; 4 Rossenstein &Oster, 1988, measure facial expression and sucking, stimuli: water [37]; 5 Crystal & Bernstein, 1998, measure: facial expression, intake, stimuli: salt water [51]; 6 Beauchamp, Cowart, Mennella, Marsh 1994, measure: sucks and intake, stimuli: salt water [43]; 7 Stein, Cowart & Beauchamp, 2006. measure: intake, stimuli: salt water [52]; 8 Stein, Cowart & Beauchamp, 2012, measure: intake, stimuli: salt water [53].

The changes in salt preferences in the first year of life are expressed in Figure 1.

## 5. Variation in Salt Preference of Infants

### 5.1. Prenatally

Infants’ preference for salty taste varies depending on a variety of factors such as physiological triggers before birth. Studies in rats suggest that extracellular dehydration and electrolyte imbalance of the mother rat can increase the salt appetite in the offspring [54]. Extracellular dehydration (as well as a fall in NaCl concentration) causes the kidneys to release renin into the circulation where it acts as an enzyme to activate angiotensin into angiotensin I, which is converted by angiotensin converting enzyme, produced mainly by the lungs, into angiotensin II. Angiotensin II plays a key role in maintaining body fluid balance and is the main stimulus for the secretion of the hormone aldosterone [55]. Angiotensin II and aldosterone are known to induce salt appetite. Both can cross the placenta and hypothetically influence the salt appetite of the off spring [54].

Extracellular dehydration and electrolyte imbalance can be caused by severe fluid loss, such as repeated severe vomiting. Relating this to humans, infants born from mothers with severe morning sickness are shown to be more likely to prefer high salt solutions, than infants from mothers who did not, or to a lesser extent, suffered from morning sickness during pregnancy [51]. This seems to have long lasting effects, as adult offspring from mothers who experienced severe morning sickness had a higher preference for salty foods, had a higher salt use, and ate more salty snack foods, than peers whose mothers did not experience such extreme morning sickness [56]. Furthermore, severe morning sickness of the mother has been associated with low birth weight of the offspring [57]. Infants with a low birth weight are more likely to prefer high salt solutions (as measured by consumption), than infants born with a normal birth weight [52].

Physiological triggers induced by severe morning sickness, as described above, are supposedly rare. It has been suggested that although around 50% of pregnant women experience morning sickness, only 0.3 to 1% of pregnant women experience severe vomiting which could lead to dehydration [58]. So although salt taste preferences seem to be influenced by physiological triggers before birth on an individual level, the impact on a population level is likely to be minimal.

### 5.2. Postnatally

Postnatally either a severe shortage of sodium as well as an over consumption of sodium might result in an enhanced preference for salty taste. Sodium deficiency during infancy and childhood is rare, except in clinical populations [59]. However, the repeated consumption of certain chloride-deficient baby formulas during infancy is suggested to result in a hormonal state (e.g., elevated plasma aldosterone levels and renin activity), which is similar to that of sodium deficiency. The long term consumption of these formulas during infancy has been positively correlated with dietary behaviours which suggest a high preference for salty foods [60]. Along the same lines, adolescents who went through severe episodes of vomiting and/or diarrhoea as an infant, causing electrolyte imbalance, show a high preference for salty foods [61].

On the other hand, the introduction of a diet high in sodium might lead to a preference for salty foods as well. Generally, by the age of 3 to 4 months infants are introduced to solid foods, including foods high in sodium such as cereals. An Australian longitudinal study shows that when infants grow from 9 to 18 months, their sodium intake doubles, with bread and rolls being the largest contributor to total sodium intake [17]. Such increased exposure to salty foods during infancy is thought to be correlated with the infants’ increased preference for salt tasting foods [50]. Potentially delaying the introduction of salty foods to infants can lower their preference for salty taste [53]. These studies have, however, a number of short comings. Harris and Booth (1987) did a very rough measurement of infants’ consumption of sodium rich foods from which it is not possible to determine the salty taste of foods and which foods were consumed [50]. Stein and colleagues (2012) focused on specific sodium rich foods (e.g., starchy foods, salted water) [53]. None of the studies investigated the specificity of sodium exposure. That is, it remains unclear if a high preference for salty foods is related to a general liking of more intense tasting foods, or if such high salt preference specifically alters the intake of salty foods. It is also not clear if preference for salty taste as measured in one medium or food relates to the intake of a wide range of salty foods. Moreover, both studies were observational and causality can not be concluded. One of the rare, but well designed, studies to investigate the causal relationship between salt consumption and salt preferences in infants was conducted in the 1980s. In a controlled study, researchers fed infants (3–8 months) either a low (2 mmol Na/100 kcal) or high (9 mmol Na/100 kcal) sodium dense diet for 5 months, and assessed their liking for salty taste and consumption of salty foods at the age of 8 years. The results show no evidence to support the hypothesis that a high salt intake during infancy resulted in either a high salt consumption or high salt preference in childhood [62]. However, the number of infants and children tested in this study was fairly small (*n* = 27).

To summarise, there is some suggestion that infants’ preference for salty taste is influenced by physiological disturbances which are initiated by severe fluid loss by either the pregnant mother or during early infancy. In addition, feeding regimes which either trigger similar hormonal systems as sodium deficiency, as well as the introduction of salty foods, potentially increase infants’ preference for salty foods. However, it remains questionable whether the consumption of high amounts of salty foods during infancy impacts liking for salty foods beyond infancy.

## 6. Salt Taste Perception of Children

Over the past 40 years, children’s taste perception and liking have been reasonably well investigated [63]. Below we provide a review of studies focused on children’s salt taste sensitivity and liking and how these relate to health outcomes such as weight status and blood pressure. Studies investigating children’s sensitivity to salt taste might provide us with insight into the physiological development of salt taste perception, whereas children’s hedonic response to salt taste might provide us with insights into children’s food choice behaviour with respect to salty foods. Therefore, both salt taste sensitivity and liking will be reviewed below.

### Children Salt Taste Sensitivity

Unlike the difficulties of measuring salt taste sensitivity with infants, salt taste sensitivity in children can be measured rather precisely. Salt taste sensitivity can be expressed as, the detection threshold (i.e., the lowest detectable concentration of NaCl), recognition threshold (i.e., the lowest NaCl concentration at which a subject can identify salty taste), or supra-threshold (i.e., the lowest difference in concentration in NaCl—in the detectable range—that is clearly perceived by a subject) [64]. To our knowledge, studies investigating salt taste sensitivity of children only focused on detection and recognition thresholds.

In order to measure detection thresholds, different methodologies have been used. Some researchers used a range of salt solutions and paired all solutions with distilled water in a 2-Alternative-Forced-Choice test (e.g., taste two samples, of which one contains salt) [65], or a 3-Alternative- Forced-Choice test (e.g., taste three samples, of which one contains salt) [66]. During the execution of this method children received all pairs of salted and distilled water, independent of the accuracy of the answers. Other researchers used a range of salt solutions and presented them in a staircase method. In this method, an inaccurate answer leads to the presentation of a higher salt concentration and an accurate answer leads to the presentation of a lower salt concentration. The presentation mode in the staircase method has either been a 2-Alternative-Forced-Choice [67,68,69], or an alternative like “taste four samples of which one contains salt” [70] or “taste 8 samples of which 4 contain salt” [71]. Most studies found detection thresholds of around 0.02% (*w*/*v*) NaCl, with one exception which found a threshold of 0.006% (*w*/*v*) NaCl. However, the latter study was conducted with a specific clinical population [71].

As suggested by Table 1, in general the staircase method seems to find lower salt taste detection thresholds than any of the other methods. In comparison, salt threshold in adults varies widely with some studies finding a threshold of 0.01% (*w*/*v*) NaCl [72], and others a three times higher threshold at 0.03% (*w*/*v*) NaCl [73]. Only two studies compared the salt detection threshold of children to those of adults in one study design. One study found higher salt detection threshold in 10- to 19-year-olds, compared with 20- to 29-year-olds [70]. Another study only found such difference when comparing boys with women [65]. None of the studies listed in Table 1 could confirm any associations between salt taste detection threshold and salt intake. One study found a higher salt taste detection threshold for those who liked soup/stews [66], whereas others failed to see such an association [67]. In adults it is generally found that salt detection threshold as measured using water solutions are not related to liking or intake [73].

Salt taste recognition thresholds are commonly conducted with a range of salt solutions and presented one at a time. Children simply reported whether they could identify the taste [74,75,76,77]. Children’s recognition thresholds of salty taste fall about 9 times above children’s salt detection threshold and vary from 0.17 to 0.18% (*w*/*v*) NaCl. Two studies found higher thresholds, but technically speaking they did not measure thresholds because of the limited number of solutions which were offered [76,77]. In adults, salt recognition thresholds vary, as is the case with children. For example, Wise and colleagues found a salt recognition threshold of 0.08% NaCl [72], whereas Lucas and colleagues found a recognition threshold of 0.11% [73]. See Table 1 for an overview.

Salt taste thresholds might reflect biological processes in the body. Sodium sensitive channels (ENaC) have been found throughout the body, including the kidneys where they play an important role in Na^+^ regulation [25]. Furthermore, animal studies suggest that salt taste sensitivity, salt uptake by the gut and salt excretion by the kidney share similar physiological pathways [78]. Differences in salt taste sensitivity might therefore be linked to high blood pressure. In adults, it has been suggested that there is a potential link between salt taste sensitivity and high blood pressure [79,80,81,82], however such a link is not uniformly shown [83,84,85].

Several studies looked into the potential link between children’s salt taste sensitivity and high blood pressure. Bobowski et al. found that systolic blood pressure was positively associated with salt taste detection thresholds in normal weight, but not overweight/obese, children [68]. This is in line with earlier findings in a group of Spanish children [69]. Kirsten and colleagues [74] investigated 14- to 19-year-olds’ ability to detect NaCl in a water solutions (e.g., 4 mmol/L, 8 mmol/L, 15 mmol/L, 30 mmol/L, 60 mmol/L, 120 mmol/L, 250 mmol/L, 500 mmol/L, 1000 mmol/L). The median concentration at which salt taste was detected was 30 mmol/L. About one third (i.e., 36%) had a detection threshold of higher than 30 mmol/L. The mean diastolic blood pressure was higher amongst those 36% than amongst the remaining sample. The mechanism behind the association between children’s salt taste perception and high blood pressure remains unknown. Potentially, salt taste perception and high blood pressure share similar physiological mechanisms.

In summary, from the limited data available there is, to our knowledge, no strong evidence to suggest that children and adults differ in their sensitivity to salt taste. However, it needs to be noted that differences in methodologies make it difficult to compare studies. There is no evidence that children’s salt taste sensitivity is related to food consumption and some evidence that lower salt sensitivity is related to higher blood pressure in some, but not all children.

## 7. Children’s Liking of Salty Taste

Unlike young infants, children as young as 3 years of age show an adult-like rejection of salted water, but show a high liking for salted soup [46]. This suggests that in general children this age might only like salt in a food context they are familiar with. The shift from acceptance to rejection of salt in water might, therefore, be influenced by the experience children have with salt tasting foods. However, it needs to be said that some researchers have found that a small proportion of children might accept salt in water [86]. Such preference might reflect a biological driver, rather than a learned preference for salty taste.

Similar to sweet taste preferences [87], there is some evidence that children prefer higher salt concentrations than adults do. Already back in 1975 Desor and colleagues showed that 9- to 15-year-old children were more likely to prefer 2.3 g NaCl/100 mL in water than adults did [86]. Such a difference in salt preference has also been demonstrated in soups [88], popocorn [89] and broth [90].

Interestingly, similar to previous findings in adults [91], children’s liking for salt is suggested to be positively related to children’s liking for sweet [90]. This suggests that liking for high levels of salt does not exclude liking for high levels of sweetness. At least two explanations for this finding can be put forward. Firstly, the increased need for energy and minerals in stages of rapid growth might simultaneously drive salt and sweet preferences in children [90]. Secondly, repeated dietary salt consumption might drive the consumption of sugar sweetened beverages [92,93] and subsequently increase liking for salt and sweet simultaneously. Both these hypotheses need further investigation.

It is not clear why children prefer higher levels of salt in foods than adults do. As shown earlier, there is no clear evidence that children and adults differ in their sensitivity to salty taste. Moreover, it is generally found that salt taste sensitivity and salt liking are not related [73]. Because exposure to, and liking of specific foods are generally found to be positively correlated in children [94], it could be hypothesised that children are more exposed to salty foods than adults, resulting in children’s higher preference for salt taste than adults. However, large population data suggest that the sodium density of children’s and adults’ diets are similar [19]. This, however does not give a clear indication of how salty the diets of children and adults taste. Lastly, it has been suggested that children’s preference for high salty foods reflects their biological need for minerals at certain stages of growth [90]. In clinical populations it has been shown that salt preferences can be increased when there is a high loss of sodium, for example in the case of congenital adrenal hyperplasia (CAH) [95]. In this disease, a genetic mutation results in adrenal insufficiency which can lead, in its severest form, to a persistent urinary loss of sodium, known as salt-wasting. Children suffering from the severe form of CAH showed an increased salt appetite, meaning they liked salt more and used salt more often. Salt wasters added 130% to 160% more NaCl (as measured with a questionnaire) to their foods than controls. In addition, Salt wasters were more likely to lick or eat pure salt. Subsequent qualitative interviews revealed that Salt Wasters developed, from a young age, strategies to deliberately consume more salt [95,96]. However a disease like CAH is extremely rare and only occurs in about 0.007% of children [96]. There is also no evidence that children have a higher need for sodium than adults do [6].

**Figure 2:**
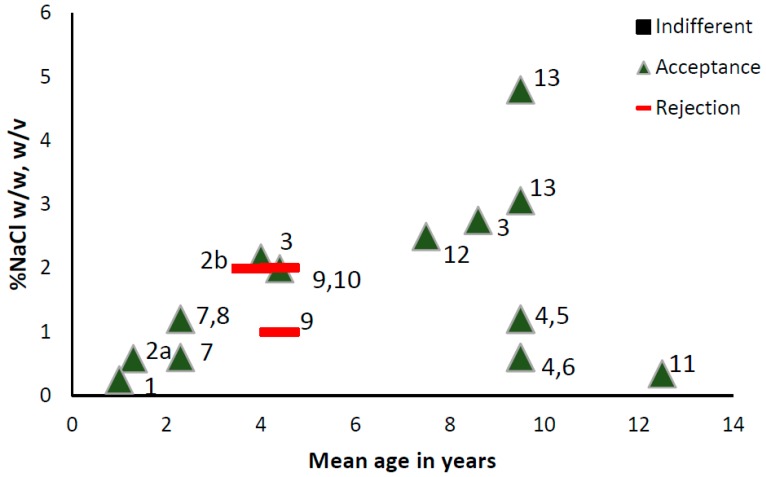
Children’s (1–13 years) indifference, acceptance and rejection responses to different concentrations of NaCl in foods (%NaCl *w*/*w*) and liquids (%NaCl *w*/*v*). 1 Harris & Booth 1987, higher consumption compared to unsalted version, mashed potatoes [50]; 2 Beauchamp & Moran 1986, higher (2a) or lower (2b) consumption compared to unsalted version, water [49]; 3 Beauchamp, Cowart & Moran 1990, most preferred, soup [88]; 4 Bouhlal, Chabanet, Issanchou & Nicklaus 2013, more liked than unsalted version, Pasta and green beans [97]; 5 Bouhlal, Chabanet, Issanchou & Nicklaus 2013, higher consumption compared to unsalted version, pasta [97]; 6 Bouhlal, Chabanet, Issanchou & Nicklaus 2013, higher consumption compared to unsalted version, green beans [97]; 7 Bouhlal, Issanchou & Nicklaus 2011, higher consumption compared to unsalted version, green beans [98]; 8 Bouhlal, Issanchou & Nicklaus 2011, higher consumption compared to unsalted version, pasta [98]; 9 Cowart & Beauchamp 1986, lower consumption compared to unsalted version, water [48]; 10 Cowart & Beauchamp 1986, higher consumption compared to unsalted version, soup [48]; 11 Kim & Lee 2009, most preferred, soup [66]; 12 Mennella, Finkbeiner, Lipchock, Hwang & Reed 2014, most preferred, broth [90]; 13 Verma, Mittal, Ghildiyal, Chaudhary & Mahajan 2007, more liked than unsalted version, popcorn (unclear statistics) [89].

Figure 2 provides an overview of the different salt concentrations children like/prefer/accept in different foods.

## 8. Children’s Salt Liking and Intake of Sodium

It is generally thought that food liking plays a key role in children’s food consumption [23]. Some studies, but not all, have been able to find a positive correlation between the liking for salt taste in adults, as tested in a controlled setting, and the consumption of sodium in everyday life [99,100]. The research into a potential association between salt taste liking and salt consumption encounters a number of challenges. Firstly, some of the sodium in the food supply cannot be tasted as being salty [30] and is mainly added to processed foods for preservation and food structural reasons [101]. Secondly, the addition of salt to foods, as mentioned earlier, influences the complete sensory profile of foods, which goes beyond making foods taste saltier [13,36]. It is therefore likely that children’s liking of added salt in one food does not necessarily translate to children’s liking of added salt in another food. Related to that, studies in adults suggest that liking for salt is food specific. That is, some foods such as salty snacks are liked when they are salty, whereas the reverse is true for foods in which salt taste deemed less appropriate [99]. Thirdly, insensitive methodology to measure children’s salt taste liking and/or salt consumption can result in a lack of correlation between salt taste liking and salt consumption. Lastly, tasting a small amount of food might not be a good predictor of consuming large amounts of the liked food [102], due to, for example, sensory specific satiety [103] or boredom [104].

The majority of studies show that the addition of salt to a variety of foods, such as soup [48,90], green beans, pasta [97,98], ricotta cheese [105], carrots [106], and popcorn [89], increases children’s liking and—if measured—consumption of that food. A large cross cultural study in 8 countries including close to two thousand children, showed that the majority of children preferred a cracker with added salt (1.6 g/100 g food) compared to a cracker with a lower concentration of salt (0.7 g/100 g food) [107]. However despite the fact that salt seems to be able to increase food liking and consumption, it does not mean that an increase in liking will always result in an increase in consumption. For example Cowart and Beauchamp (1986) showed that for half of the 3- to 6-year old children they tested, the most liked soup did not correspond to the soup they drank the most of [48]. In a study with 8- to 11-year-old it was found that small additions of salt would increase liking, but not consumption of pasta [108]. Furthermore, it is important to notice that the addition of salt does not influence children’s liking, nor intake equally across different foods [105,108]. Hypothetically, children like the changed flavour profile of the foods, as a result of the addition of salt, rather than salt taste itself.

Potentially, liking of salty foods is related to small increases in salt consumption which cannot be measured in single foods, but can be measured when focusing on daily sodium consumption. A study which measured 5- to 10-year-old children’s liking for salt (0.92–6.14 g NaCl/100 g of broth) in broth, found that liking for salt in broth was positively related to daily sodium intake (r = 0.24). It was, however, not reported which foods contributed most to the daily sodium consumption [90]. The latter is important because the sodium content of food does not necessarily mean they taste salty. So it is likely that children’s salt preference is correlated with some foods, but not others. Kim et al. suggested that salt preference as measured in soup was positively associated with the frequent consumption of certain salty foods (i.e., pork cutlets and hamburgers), but not with other foods (e.g., pizza, fried chicken) [66]. If one assumes that there is some positive relationship between children’s salt taste liking and intake of salty foods, one might also expect an association between children’s salt taste liking and health outcomes such as weight status and high blood pressure. However, to our knowledge such associations have not been found [107,109]. This supports the view that children’s salt taste preferences are unlikely to have a generic effect on children’s diet.

All these studies are, however, observational studies, and causality cannot be concluded. How modifiable are children’s salt preferences? Repeated exposure to a salted food has been shown to increase children’s liking for that particular salted food, but not for a salted food to which the child was not exposed [105]. Another study confirmed that repeated exposure to salted foods increases intake of that food, but it is not clear if such exposure leads to a high liking for salt taste [108]. These studies suggest that liking for a particular salted food can be changed by repeated exposure. However, it does not support the hypothesis that repeated exposure to salty foods increase children’s generic liking for salty taste.

In adults, salt taste liking can be shifted downwards by exposing individuals to low sodium diets. Such shifts have been observed in randomized controlled studies in which adults were placed on a low sodium diet for 2 weeks to several months (see [110] for review). Not only liking for salt taste can be adjusted downwards, but salt taste intensity can be increased as a result of a low sodium diets which is maintained for at least 2 months [111]. For an extensive review on this topic see [15]. It is important to note that all these intervention studies applied an overall reduction of sodium in the diet, rather than a sodium reduction in one single food. To date it remains unclear if a reduction in one single food would result in an overall liking of reduced levels of sodium in a variety of foods.

One can speculate that by repeated exposure, children become familiar with foods. This familiarisation can drive liking [94]. However, to our knowledge, there are no experimental studies carried out with children to verify if a repeated exposure to either low salty or high salty food can modify children’s generic liking for salt.

In summary, there is some evidence that children, compared to adults, prefer higher concentrations of salt in soups and crackers. However, there seem to be no studies that investigated if children, compared to adults, prefer higher concentrations of salt in a variety of foods, such as salt-fat foods, meats, and vegetables, than adults. Most studies suggest that the addition of salt to foods increases children’s liking for the salted food, however it is unlikely that such liking represents a generic liking for salty taste. The evidence for a positive association between the liking of a salty taste and ingestion of salty foods in general is not convincing. It is also worth noting that although children seem to have a liking for higher concentrations of salt than adults, such a difference does not manifest itself in a difference in the sodium density of children’s and adults’ diets [19].

## 9. Conclusions

The high consumption of sodium by children is worrisome and a better understanding of what might contribute to this high consumption might aid to the development of strategies to decrease this high sodium consumption. The present review highlights the role of taste in infants’ and children’s consumption of high sodium foods.

The current review suggests that both biology and learned experiences influence infants’ and children’s liking for salty foods. Although the liking of salty taste starts as an unlearned response in early infancy, this liking soon develops as a result of repeated exposure to salty foods. The available studies seem to suggest that infancy is a potentially sensitive period in which salt taste preferences could be modified. Generally speaking, a low exposure to salty foods is associated with a low preference for salty foods. Randomised controlled trials, however, are needed to provide clarity about the causality of such relationship. No study, to our knowledge, suggested that decreasing the exposure to salty foods during infancy is associated with an increased liking or desire for salty foods. Therefore, limiting infants’ consumption of salty foods to decrease sodium consumption and potentially decrease liking for salty foods seems to be a sensible approach. At the same time it is not recommended to try to eliminate sodium from the infants’ diet all together, because severe sodium deficiency has been linked to an increased liking of salty taste amongst other medical complications. The role of repeated exposure to salty foods during infancy and subsequent liking of salty taste and consumption of salty foods during childhood, adolescence and adulthood requires, however, more systematic research. Randomised controlled trials are needed to provide insight into whether avoiding high sodium foods during infancy can have a long-lasting effect on the development of salt taste liking and the consumption of salty foods.

Several studies showed that children have a preference for a higher level of saltiness than adults. Such heightened preference does not seem to result in a general diet higher in sodium density. However, studies in which salt is added to a variety of foods have consistently shown that children’s food consumption can be increased when salt is added. The amount of salt which needs to be added to significantly increase consumption is, however, food dependent. This seems to suggest that it is not salt taste per se which drives consumption, but the effect salt has on the complete sensory profile of foods which drives consumption. To date there is a lack of research investigating mechanisms by which a change of salt content can modify children’s liking, desire and intake of a variety of foods.

Hypothetically, by slowly decreasing the amount of salt in specific foods children consume, one might be able to decrease children’s liking for these specific salty foods as has been suggested in adults. However, such strategy should include the whole diet rather than single foods and needs to be informed by randomised controlled trials. A similar strategy has been suggested for adults [15].

In conclusion, decreasing exposure to salty tasting foods during early infancy is recommended. Salt plays an important role in children’s liking for a variety of foods. It is questionable whether children’s liking for salt per se influences their intake of salty foods.

## Figures and Tables

**Table 1:** Children’s salt taste detection and recognition threshold.

Population *N*, Age Range, Country	Type of Threshold (Design)	Solution Range %NaCl in Water	Threshold	Remarks		Reference
*N =* 251, 10–12 years, Japan	Detection (filter paper, paired comparison)	0.6–1.6	0.6%	0.6%NaCl was lowest concentration presented.	Thresholds not related to liking or salt intake	Matsuzuki et al., 2008 [67]
*N =* 24 10–19 years , UK	Detection (staircase, one in 4)	0.004–0.58	0.04%	Mean based on interpretation of figure	Threshold 10–19 years old is higher than 20–29 years old	Baker et al., 1983 [70]
*N =* 70, 12–13 years, Korea	Detection (triangle test)	0.005–0.15	0.03%		Higher thresholds for those liking soup/stew	Kim and Lee, 2009 [66]
*N =* 97, 8–14 years, USA	Detection (staircase, paired comparison)	0.0003–5.8	0.021%	52% overweight children	Threshold not related to salt intake	Bobowski & Mennella, 2015 [68]
*N =* 68, 8–9 years, Australia	Detection (paired comparison)	0.0009–0.029	0.016–0.036%	Boys were less sensitive than adults	Boys had higher threshold than women	James, Laing, & Oram, 1997, [65]
*N =* 72, Age unknown, Spain	Detection (staircase paired comparison)	0.0012–0.08	0.027%			Arguelles et al., 2006 [69]
*N =* 22, 9–19 years, USA	Detection (staircase 4 in 8)	0.00006–5.8	0.006%	Clinical population		Hertz et al., 1975 [71]
*N =* 421, 14–19 years, Brazil	Recognition	0.02–5.8	0.17%		Threshold not related to body composition	Kirsten & Wagner, 2014, [74]
*N =* 237, 6–15 years, Japan	Recognition (one solution)	-	0.4%	0.4% was the only solution presented	Sensitivity lowest in 4–6 graders	Ohnuki et al., 2014 [77]
*N =* 40, 5–12 years, Italy	Recognition (two solutions)	0.18, 1.8	1.8%	Only two solutions tested		Majorana et al., 2012 [76]
*N =* 319, 9–17 years, Nigeria	Recognition threshold (range)	0.18–1	0.18%		Higher threshold in boys than girls	Okoro et al., 1998 [75]
